# Supplementary figures and images for: Identification and serological responses to a novel Plasmodium vivax merozoite surface protein 1 (PvMSP-1) derived synthetic peptide: a putative biomarker for malaria exposure
Source: PeerJ. 2024 Jun 25;12:e17632. doi: 10.7717/peerj.17632 (PMC11212635; doi:10.7717/peerj.17632)

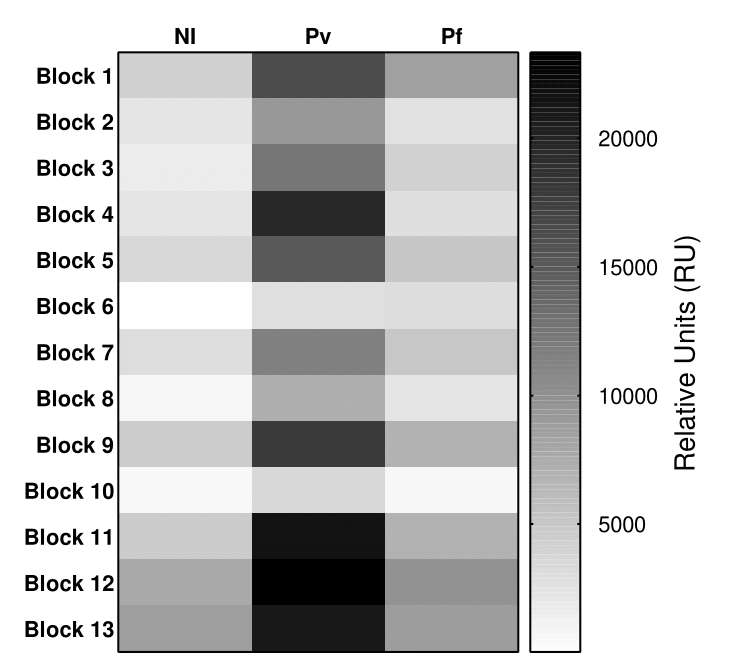

Supplement: Supplemental Information 1 — A heatmap was generated using the mean relative intensity for conserved blocks (1, 3, 5, 7, 9, 11, and 13) and polymorphic blocks (2, 4, 6, 8, 10, and 12). NI, non-infected individuals; Pv, P. vivax-infected individuals; Pf, P. falciparum-infected individuals. [file peerj-12-17632-s001.png]

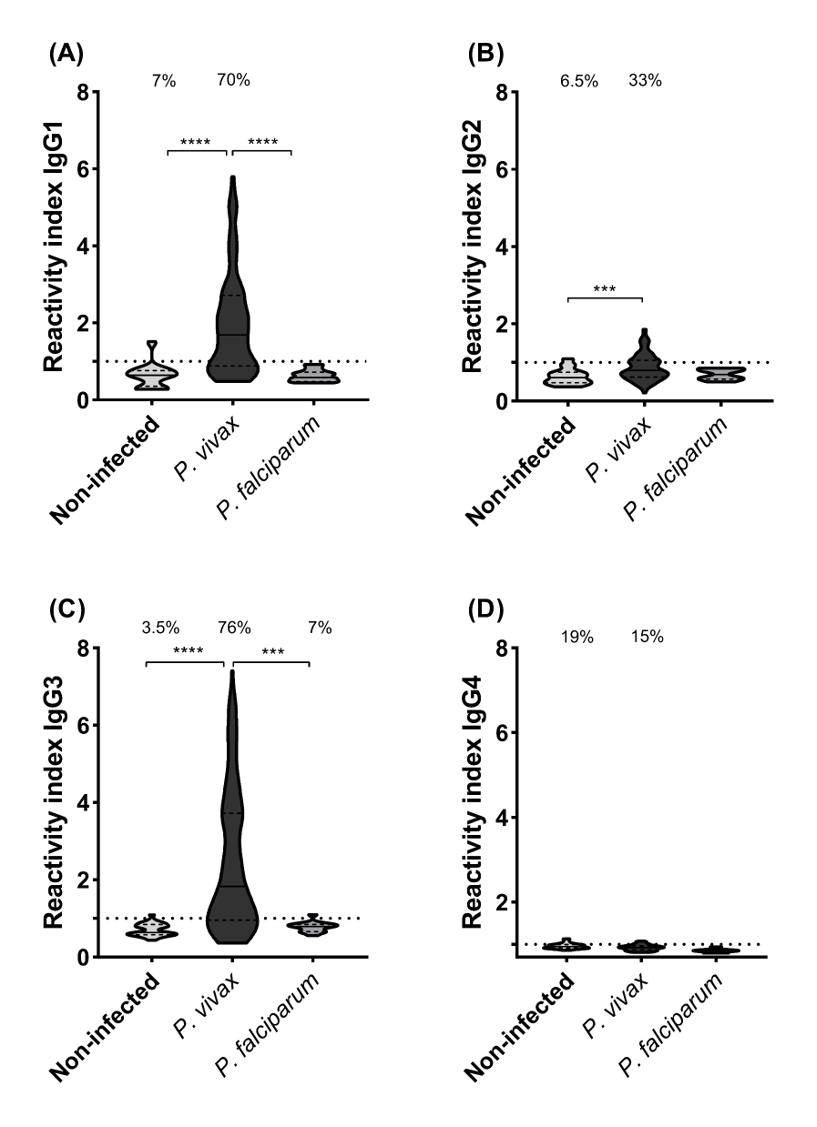

Supplement: Supplemental Information 2 — IgG subclass levels were determined using ELISA. Panels (A) through (D) represent IgG1, IgG2, IgG3, and IgG4, respectively among healthy donors (n=35), P. vivax patients (n=152), and P. falciparum patients (n=35). Antibody levels are expressed as Relative Intensity (RI). The violin plot illustrates interquartile intervals and the median. Percentage values indicate the overall frequency of positive responders within each group. Statistical significance was evaluated using the Kruskal-Wallis test followed by Dunn’s post-hoc test (*p ¡ 0.05; **p ¡ 0.01; ***p ¡ 0.001; ****p ¡ 0.0001). [file peerj-12-17632-s002.png]
