# Supplementary material for: Identification and serological responses to a novel Plasmodium vivax merozoite surface protein 1 (PvMSP-1) derived synthetic peptide: a putative biomarker for malaria exposure
Source: PeerJ. 2024 Jun 25;12:e17632. doi: 10.7717/peerj.17632 (PMC11212635; doi:10.7717/peerj.17632)
Supplement: Supplemental Information 3 [file peerj-12-17632-s003.docx]

| *Plasmodium vivax* Merozoite Surface Protein-1  peptide 314 | | | | | | |
| --- | --- | --- | --- | --- | --- | --- |
| **Score** | **E-value** | **Identity** | | **Positivity** | | Gaps |
| 58.2 (117) | 2e-09 | 15/15 (100%) | | Consult  1 ETKCDELDLLFNVQN 15  *Pv*MSP-1 880 ETKCDELDLLFNVQN 895 | | 0/15 (0%) |
| **Organism** | | | **Number of Hits** | | Alignment | |
| *Plasmodium vivax* | | | 139 | | 100% | |
| *Plasmodium vivax* North Korean | | | 2 | | 100% | |
| *Plasmodium vivax* Indian VII | | | 1 | | 100% | |
| *Plasmodium vivax* Brazil | | | 1 | | 100% | |
| *Plasmodium vivax* Merozoite Surface Protein-1  peptide 70 | | | | | | |
| **Score** | **E-value** | **Identity** | | **Positivity** | | Gaps |
| 52.8 (117) | 2e-06 | 15/15(100%) | | Consult  1 KIPEHLKISDKELDM 15  *Pv*MSP-1 148 KIPEHLKISDKELDM 162 | | 0/15 (0%) |
| **Organism** | | | **Number of Hits** | | Alignment | |
| *Plasmodium vivax* | | | 143 | | 100% | |
| *Plasmodium cynomolgi* | | | 12 | | 100% | |
| *Plasmodium coatneyi* | | | 3 | | 100% | |

**Supplemental Table 1.**

**Similarity between Plasmodium spp. MSP-1 sequences and peptides p314 and p70**
